# Spatial Optimization of Future Urban Development with Regards to Climate Risk and Sustainability Objectives

**DOI:** 10.1111/risa.12777

**Published:** 2017-02-23

**Authors:** Daniel Caparros‐Midwood, Stuart Barr, Richard Dawson

**Affiliations:** ^1^ School of Civil Engineering and Geosciences Newcastle University UK

**Keywords:** Climate risks, genetic algorithm, spatial optimization, sustainability objectives, urban planning

## Abstract

Future development in cities needs to manage increasing populations, climate‐related risks, and sustainable development objectives such as reducing greenhouse gas emissions. Planners therefore face a challenge of multidimensional, spatial optimization in order to balance potential tradeoffs and maximize synergies between risks and other objectives. To address this, a spatial optimization framework has been developed. This uses a spatially implemented genetic algorithm to generate a set of Pareto‐optimal results that provide planners with the best set of trade‐off spatial plans for six risk and sustainability objectives: (i) minimize heat risks, (ii) minimize flooding risks, (iii) minimize transport travel costs to minimize associated emissions, (iv) maximize brownfield development, (v) minimize urban sprawl, and (vi) prevent development of greenspace. The framework is applied to Greater London (U.K.) and shown to generate spatial development strategies that are optimal for specific objectives and differ significantly from the existing development strategies. In addition, the analysis reveals tradeoffs between different risks as well as between risk and sustainability objectives. While increases in heat or flood risk can be avoided, there are no strategies that do not increase at least one of these. Tradeoffs between risk and other sustainability objectives can be more severe, for example, minimizing heat risk is only possible if future development is allowed to sprawl significantly. The results highlight the importance of spatial structure in modulating risks and other sustainability objectives. However, not all planning objectives are suited to quantified optimization and so the results should form part of an evidence base to improve the delivery of risk and sustainability management in future urban development.

## 1. INTRODUCTION

Urbanization and the increased frequency of climate‐change‐induced extreme events are driving a move to designing increasingly resilient cities globally.^1^, ^2^ The historical development of urban areas has led to a spatial form that is poorly adapted to hazards^3^ while major cities are frequently located within high‐risk areas such as coastal zones.^4^ Increasing urbanization, reaching 60% of the world's population by 2030,^5^ will only exacerbate the risks and vulnerability of cities to more frequent extreme weather events due to climate change.^1^, ^2^, ^6^ Consequently, national and local governments are considering their adaptation options in cities^7^, ^8^ and considering how these risk might be accounted for within future urban development in order to alleviate the potential effects of extreme events on their populations and infrastructure.^9^

However, risk management does not exist within a policy vacuum. Policies are also being enacted to reduce the greenhouse gas emissions of energy and transport systems in accordance with global efforts to mitigate the drivers of climate change.^10^ Legislation and policies, which in the United Kingdom include the 2008 Climate Change Act,^11^ place binding targets on policymakers to reduce greenhouse gas (GHG) emissions (reduction in CO_2_ of 26% by 2020 and 80% by 2050 against a 1990 baseline in the United Kingdom). Much of this reduction will have to be delivered in cities, which make the largest contributions to energy use.^12^ Thus, cities around the world are the “front line” for reducing energy and resource usage while reducing risk from climate‐change‐induced hazards.^7^, ^12^

Simultaneous pursuit of these aspirations for cities has the potential to create conflicting outcomes. For example, the policy of urban intensification, intended to reduce transport energy use,^8^ can negatively affect risk management efforts because reduced surface permeability increases flood risk and the proximity of buildings can intensify urban heat islands.^2^, ^13^ Conversely, some adaptation efforts have been found to have increased greenhouse gas emissions;^14^ for example, the use of desalination plants to secure water supplies and air conditioning to combat heat stress are both energy intensive. Moreover, these unintended outcomes often disproportionately impact the most vulnerable.^15^, ^16^, ^17^ The lack of a whole systems approach to understanding the interaction of these risks and objectives can lead to well‐intended interventions in one sector causing negative outcomes in another.^18^ There is therefore a need for planners to make decisions based on locally specific evidence, across a range of sectors, rather than applying one‐size‐fits‐all policies.^18^ This requires more sophisticated methods of analyzing risk and sustainability objectives, such that coherent planning decisions can be made that can subsequently be implemented by key stakeholders, such as developers and utility operators.

In this regard, there is an increasing awareness of the potential risk to long‐term coherent urban planning of cities due to climate‐related hazards.^19^ A number of previous studies have focused on developing methodologies to assess the economic impact of future flooding,^20^ human mortality from increased heat wave frequency,^21^ and the resilience of urban infrastructure to natural disasters.^22^ Such approaches are useful to demonstrate the impacts of potential hazards. However, they often lack the ability to provide information on how to best maximize desirable outcomes with regard to the hazard in question and rarely are able to provide ranked alternative strategies or planning pathways to aid decision making.^23^ This is crucial if the aim is to better inform the urban planning process.^24^ Moreover, they are often limited to a single hazard or sustainability objective (i.e., climate risk, emissions, employment)^25^ when it is recognized that multiple hazards and sustainability elements need to be considered simultaneously due to their often complex relationships and interactions.^18^

In this context, a growing body of work has demonstrated that optimization techniques can be successfully employed to provide optimal infrastructure plans in the presence of multiple objectives. These include planning of water distribution networks,^26^, ^27^ design of bus transport networks,^28^, ^29^ and planning of land use.^30^, ^31^ In the case of land use, where studies have considered sustainability in a spatial context they have focused almost exclusively on the compactness and compatibility of land use premised on the hypothesis that compact cities are more sustainable.^30^, ^32^, ^33^, ^34^, ^35^ To the authors’ knowledge no previous studies have considered adaptation to risks alongside the sustainability objectives that focus more on energy use and greenhouse gas emissions. However, as already noted, the pursuit of one sustainable constituent to the exclusion of others has a high potential for causing negative consequences in other elements. Moreover, applications to sustainable urban planning studies have been limited to synthetic urban areas,^33^, ^36^ small urban towns,^30^, ^35^ and regional areas,^31^, ^37^ with little evidence of successful implementation in large urban areas. This is unfortunate as the literature indicates that major metropolitan areas face the greatest risk management, mitigation, and adaption challenges today and into the future, and therefore must be a focus of long‐term sustainable planning efforts.^7^

With regard to particular optimization approaches, genetic algorithms (GAs) are increasingly utilized over traditional optimization approaches, such as linear programming and simulated annealing, due to their improved ability to find globally (in terms of the search space) optimal solutions coupled with shorter search times.^38^, ^39^ For this reason, they have been utilized in a number of spatial optimization studies.^31^, ^40^, ^41^ A major advantage of GA is its ability to handle multiobjective optimization through Pareto optimization, whereby a number of mathematically determined optimal solutions that are best tradeoffs to a problem are returned. Studies have found this approach to be particularly suitable for multiobjective decision making^42^ and it has been described as an ideal method by which to present the results of optimization of urban planning^43^ and sustainability applications.^23^ However, despite this there is a dearth of applications that determine tradeoffs in the field of sustainable urban planning. The majority of spatially explicit applications focus on utilizing GA to approaches to identify individual spatial development configurations that best optimize a single objective^44^ or utilize a weighting system to identify a limited number of optimal spatial development configurations based on prior preferences.^33^, ^41^ This weighting approach is preferred as results are returned in a shorter time frame (compared to Pareto optimization, which is both time and computationally intensive^45^). However, within such an approach the full spectrum of diagnostic information on the interactions and conflicts between the objectives is lost. As discussed earlier in this section, an understanding of these interactions is crucial in order to help planners meet the challenges of complementing risk management strategies with other strains of sustainability.

Therefore, in this article we outline a developed spatial optimization framework powered by a GA and Pareto optimization to help decisionmakers identify optimal spatial planning strategies of cities in the presence of multiple, conflicting risk and sustainability objectives. The framework is applied to a case study to planning future development at a fine spatial scale in London, a major foci for sustainable urban development in Europe, and works toward a number of highly prioritized risk and sustainability objectives alongside current planning policies to demonstrate its applicability to real‐world planning. Following this case study, the implications of the results and potential application of the optimization framework are considered.

## 2. METHODOLOGY

### 2.1. Selection of Risk and Sustainability Objectives

To understand the current real‐world sustainable planning pressures an extensive review was undertaken of the current U.K. government adaptation policies^46^, ^47^ as well as spatial planning literature.^2^, ^9^, ^13^ Additionally, local sustainability appraisals and current planning policy were analyzed.^48^, ^49^, ^50^, ^51^ From this review a series of highly prioritized risk and sustainability objectives were collated for use in the proposed spatial optimization algorithm for which data are known to exist for the United Kingdom and that could be represented in the form of spatial fields. The finalized set of objectives selected for analysis within the framework were:

- **Minimizing exposure to future heat wave events**: Appeared in 40% of sustainability appraisals reviewed and prioritized by national governments, including the United Kingdom.^46^
- **Minimizing risk from future flood events**: Highly prioritized by 70% of sustainability appraisals reviewed and a priority policy for the U.K. government.^47^
- **Minimize travel costs to minimize transport emissions**: All sustainability appraisals reviewed stated this as a high priority objective.^48^
- **Maximizing brownfield development**: A national government planning policy objective is to maximize the development to brownfield sites in order to limit unnecessary greenspace development.^52^
- **Minimizing the expansion of urban sprawl**: A national priority through policies encouraging development on previously developed sites within existing urban areas.^53^
- **Preventing development of greenspace**: Appears as a sustainability objective in 80% of sustainability appraisals reviewed.

### 2.2. Problem Formulation

As with previous applications the urban system is spatially represented as a raster‐gridded data set.^37^, ^41^ A proposed spatial development plan is defined as an array *D* indexed by *l*, which corresponds to a location in the study area with a coordinate i,j. Assigned residential development sites within the study are defined as *d* and a collection of these form a development plan *D*, noting that a number of *l* can remain undeveloped, for example, $D\;=\;[0,d_{1},d_{2},\;0,\;0\ldots ]$. Assigned residential development, $d_{l}$, are allotted a density den; thus the number of dwellings associated with each development is ddw=area∗d den . To form a feasible development plan, the following constraint ensures that a required number of dwellings are assigned:

(1) SubjecttoDw MIN ≤Ddw≤Dw MAX ,

where Dw MIN  and Dw MAX  represent minimum and maximum possible number of dwellings in a development plan and $D^{dw}$ represents the total number of dwellings associated with a particular development plan (i.e., the sum of $d^{dw}$). This allows the GA to fully investigate the objective space. As the total number of new dwellings and their density can vary between these bounds, the objective functions (Equations (2)–(6)) are all in proportion to the value of $D^{dw}$.

***Objective (i)*** was minimized on the basis of the objective function *f*_heat_ defined as:

(2) $$\mathrm{Min}\left(\sum h_{l}d_{l}^{dw}\propto D^{dw}\right),\;$$

where *h* refers to a heat hazard value, here defined in terms of the number of heat wave days where temperatures exceed 32 °C,^54^ at the location identified by *l*. On the basis of this, the framework aims to prevent appropriating development in areas with high incidence of heat wave hazard identified from the observations of the UrbClim heat wave model.^55^ While the calculation does not consider an increase in the urban heat island resulting from the placement of new dwellings, the urban heat island produced by the UrbClim model includes existing development density in its derivation and a large increase in urban development is required to substantially alter the heat island properties.^56^

***Objective (ii)*** is optimized on the basis of the objective function *f*_flood_, which is characterized by a flood risk assessment of development that occurs within the 1 in 100 and 1 in 1,000 year floodplain zones. These two zones are the thresholds used in the planning process and are set by the U.K. government in its Planning and Policy Statement on Development and Flood Risk.^57^ Flood risk is a combination of likelihood and impact, so is calculated here in terms of the amount of development in each zone weighted according to the relative likelihood of flooding. As such flood risk is represented as:

(3) Min 100∑z100dldw+10−1∑z1000dldw∝Ddw,

where *z* ^100^ and *z* ^1000^ are spatial grids representing the 1 in 100 and 1 in 1,000 flood zone extents, respectively.

***Objective (iii)*** is optimized on the basis of an accessibility measure of new development to areas of employment and services, characterized by the distance of proposed development to a town center. The optimization attempts to minimize the objective function *f*_dist_, which is expressed as:

(4) Min Pdl,cl,R∀cl∧dl∈D∝Ddw,

where *P*() is the shortest path between a $d_{l}$ and it is the closest point designated as a town center centroid, $c_{ij}$, over a road network, *R*.

***Objective (iv)*** is optimized on the basis of the objective function *f*_brownfield_, which attempts to minimize the number of proposed development sites that do not fall in cells designated as brownfield sites, $b_{l}$:

(5) $$\mathrm{Min}\left(\sum d_{l}\neq b_{l}\;\forall \;d_{l}\in D\propto D^{dw}\right).\;$$

***Objective (v)*** is parameterized as a minimization of the number of proposed development sites falling outside the current developed urban land $u_{l}$ to prevent urban sprawl. This is represented by the objective function *f*_sprawl_:

(6) $$\mathrm{Min}\left(\sum d_{l}\neq u_{l}\;\forall \;d_{l}\in D\propto D^{dw}\right).\;$$

***Objective (vi)*** is enforced through a spatial constraint that prevents the appropriation of development to cells designated as greenspace, $g_{l}$:

(7) $$Subject\;to\;\;\;\;\;d_{l}\;\neq g_{l}\forall \;d_{l}\in D.\;$$

A final constraint ensures development is only possible in cells that have available space for development:

(8) $$Subject\;to\;\;\;\;d_{l}=\;1\;\;\mathrm{if}\;d_{l}\cap \;a_{l},\;$$

where $a_{l}$ represents cells designated as being available for development (also known as active cells).

The final objective performances of spatial strategies are normalized to enable comparison of different measures, for example, the increased vulnerability of people to heat against damages to property from flooding. Normalized objective values were calculated for each development strategy using:

(9) fs norm =fs−fs min /fs max −fs min ,

where fs min  and fs max  represent the maximum and minimum performance for each objective function, *f*. This enables discussions on relative tradeoffs between objectives, and debate about the relative societal importance of different objectives, to be disentangled. Evidently, where the absolute range of each objective is small, the value of optimization is limited.

### 2.3. Spatial Optimization Using a GA

#### 2.3.1. Implementation of the Spatial GA

**Figure 1:**
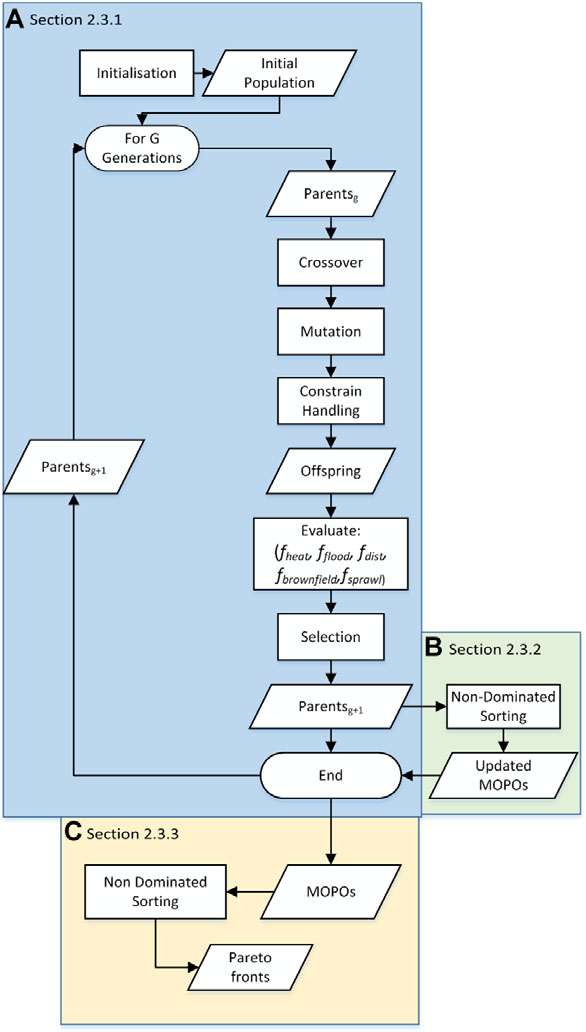
Flow diagram of the Genetic Algorithm Spatial Optimization Framework, separated into key steps (a–c) described in Sections 2.3.1.–2.3.3.

Fig. 1 shows the structural components of the GA approach. Fig. 1(a) demonstrates the initialization phase consisting of producing an initial set of development plans of size No parents , the majority of which are randomly generated. However, to aid convergence a minority of initialized development plans are biased to certain variables and extremes on the basis of a small prior probability. For example, a small number of the initial set is restricted to brownfield or a single development density. This preferential initialization helps accelerate the convergence of spatial plans to being optimal across the sustainability objectives. The initialization therefore provides an initial set of parent spatial plans for the evolutionary operators to modify.

Thereafter, Fig. 1(a) shows the steps for applying the genetic operators. At each generation, *g*, for a defined number of generations, *G*, the GA operators of crossover, mutation, and selection are applied to $parent_{g}$ solutions to produce a next generation, $parents_{g+1}$. The crossover operator exchanges the attributes in *D* of pairs of solutions, based on a probability *p*_crossover_, around two randomly selected crossover points producing two newly produced spatial plans that are potentially superior. Next, the solutions are subject to a mutation operator based on a probability *p*_mutation_ and their elements mutated based on the probability, $p_{m}$. In this work, the framework utilizes a shuffle index mutation where selected elements are swapped within *D*. This retains the original *D* ^dwells^ while spatially varying the allocation of *d*. The purpose of the mutation operator is to maintain diversity in the offspring and prevent premature convergence on a set of $d_{l}$.

At this point in the operation constraints are applied to the newly produced set to ensure they are feasible spatial plans that meet Equations (2), (7), and (8). Following previous recommendations,^58^ Equations (2) and (8) are handled by restricting the variable space to consider only solutions that are in $a_{ij}$ (i.e., areas available for development) but not in $g_{ij}$ (i.e., areas not designated as greenspace). Equation (7) is enforced through discarding of infeasible spatial plans where solutions do not meet the prescribed number of dwellings, while solutions that do are retained to form a set of *offspring* solutions. These are combined with the parent solutions before a selection operator extracts superior solutions to form $\mathrm{parent}s_{g+1}$ mimicking natural selection in evolution where the strongest survive and proceed to produce further offspring. This work utilizes a selection operator based on the NSGA‐II selection procedure^59^ to extract the most optimal solutions at each iteration. NSGA‐II has been proven for other spatial optimization applications^30^, ^60^ and is more efficient than many other algorithms as its computational complexity is proportional to the square of the population size, as opposed to the cube.^59^ The definition in Section 2.3.2. is used to identify the Pareto optimality of solutions in $parent_{g}$ and offspring while a measure of how diverse each solution is with respect to the other solutions is calculated. These two factors are then used to reduce $parent_{g}$ and offspring to $parents_{g+1}$ ensuring the most optimal are carried through and preserving a diverse representation.^59^

The steps outlined in Fig. 1(a) are repeated for *G* number of times, each time producing a new set off solutions at each generation. Selected solutions from the offspring and parent sets replace the $parent_{g}$ solutions at each generation. This way the search space is gradually explored culminating in the best known solutions remaining.

#### 2.3.2. Pareto Optimization

Throughout the operation, the algorithm strives toward identifying the Pareto‐optimal set of solutions to the planning problem. A Pareto‐optimal solution in optimization is defined as a solution that outperforms all other solutions in at least one objective and is based on the concept of domination.^61^ For *F* objective functions a solution *s* ^(1)^ is said to dominate solution$\;s^{\left(2\right)}$ if:

(1) The solution *s* ^(1)^ is no worse than *s* ^(2)^ in all objectives; $f\left(s^{\left(1\right)}\right)\leq f\left(s^{\left(2\right)}\right)\forall \;f\in F$.
(2) The solution *s* ^(1)^ is strictly better than *s* ^(2)^ in at least one objective; $f\left(s^{\left(1\right)}\right)<f\left(s^{\left(2\right)}\right)$ for at least one fεF ^61^.

This process of Pareto‐optimization is shown in Fig. 1(b) where at the end of each g∈G newly found solutions are assessed against the existing Pareto‐optimal set, *N*, through a process called nondominated sorting. If a solution, $s^{n}$, is found to dominate a solution in *N*, it is added to *N*, and the solution (*s*) in *N* dominated by $s^{n}$ is removed. This ensures that *N* comprises the best set of Pareto‐optimal sets of solutions found throughout the search. During the GA application, domination is based on the entire set of objectives and as such the resulting set is referred to as multiobjective Pareto‐optimal solutions (MOPOs). This set is returned upon completion of the algorithm.

#### 2.3.3. Pareto‐Optimal Solution Sets

Fig. 1(c) shows the processing of outputs from the GA once it has completed *G* generations. The MOPO solution set represents the Pareto‐optimal spatial configurations where no other spatial configuration performs better for the combination F=f heat ,f flood ,f dist ,f brownfield ,f sprawl . However, in order to further understand the conflicts and interactions between pairs of objectives, Pareto‐optimal sets were extracted from the MOPO set for different combinations of *F*.

For example, {f heat ,f flood }⊆F,{f heat ,f dist }⊆F…to produce Nf heat ,f flood , Nf heat ,f dist …etc.

These provide Pareto‐optimal sets between objectives and, when plotted against the objectives, present the best trade‐off curve, referred to as the Pareto front between the objectives of interest. The nondominated sorting procedure outlined by Mishra and Harit^62^ was used to perform this operation by initially ranking the MOPO set ascendingly by the first objective, *f*_1_. Next, the top solution is popped into $N_{F}$ then in descending order the sorted solutions as compared against $N_{F}$, updating it as per nondominated sorting. The approach has been shown to reduce the number of computations compared to other nondominated sorting procedures as dominated solutions are identified and disregarded more quickly.^62^

The framework described here was developed in the Python programing platform and utilized the software package Distributed Evolutionary Algorithms in Python (DEAP)^63^ to support the implementation of the GA operators (selection, crossover, and mutation).

## 3. LONDON CASE STUDY

### 3.1. Case Study Description

**Figure 2:**
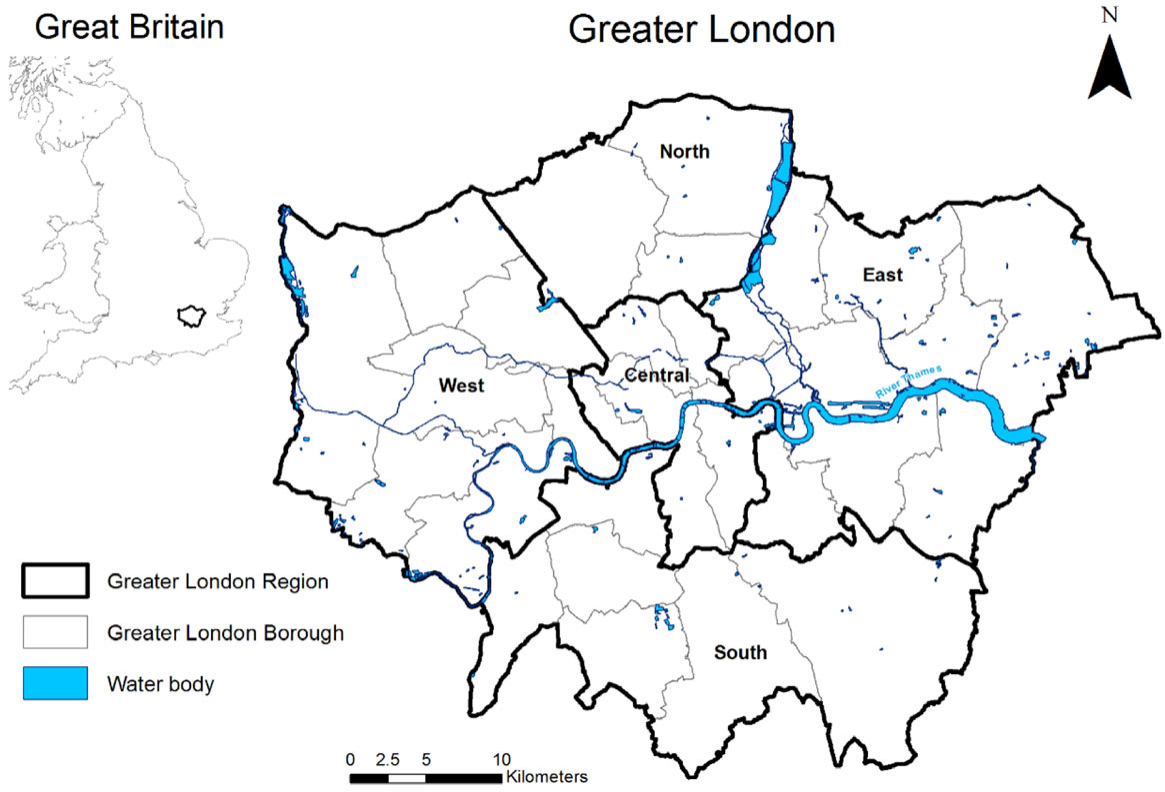
Greater London study area.

To demonstrate the utility of the developed spatial optimization framework it was applied to the problem of determining future residential development in Greater London, shown in Fig. 2, an area of 1,572 km^2^. London is experiencing pressures from a high degree of population growth while simultaneously facing increased future heat waves (exacerbated by heat islands) and higher risk of flooding from the Thames and its estuaries due to climate change.^64^, ^65^ Meanwhile, London has set itself ambitious CO_2_ emission reductions of 60% (below 1990 levels) by 2025.^66^ The case study considers the residential development priorities and plans up to 2021 that are set out in the Greater London Authority's (GLA) Spatial Development Strategy.^48^ In particular, the strategy sets out a focus on development in east London with 25% of all proposed new dwellings planned for just three east London boroughs (of 33 boroughs in total). The strategy also identifies key development locations that are centered around a series of suburban hubs within London itself, referred to as “London's town center network.”^48^ This development strategy is compared with results from the spatial optimization framework.

#### 3.1.1. Problem Definition

Figures for Dw MIN  and Dw MAX  were derived from the Greater London's Spatial Strategy's^48^ sustainability target of 322,100 net additional dwellings over a 10‐year period 2011–2021 (32,210 per annum) and their 340,000 estimated requirement to accommodate population growth for the same 10‐year period (34,000 per annum).^48^ To constrain the search space, a set of development densities were derived (from Table 3.1 in London's Spatial Strategy^48^) that capture lower and upper bounds of real development, and sensible interim values, den={35,60,100,150,250,400} where each figure is in units (dwellings) per‐hectare (uha). A spatial resolution of 200 m (cell size of 40,000 square meters or 4 hectares) was found to provide a suitable balance between computational expense and spatial resolution. The number of dwellings that can be assigned to each cell is therefore four times the density, dw={140,240,400,600,1,000,1,600}.

#### 3.1.2. Local Contextual Constraints

In order to comply with current planning policy in London a further constraint was added to the spatial optimization to ensure proposed development densities meet the Public Transport Accessibility Layer (PTAL) standards for accessibility (Table I). This ensures that high densities of development occur in high accessibility PTAL areas derived on the basis of the density of the public transport network at any location. Spatial plans that are generated by the GA, but do not meet this constraint, are automatically discarded.

**Table I:** Public Transport Accessibility Layer (PTAL) Accessibility Standard for New Development in London (Adapted from Table 3A.2 in London's Spatial Strategy^48^)

PTAL Classification (see Fig. 3f)	1a (Low Accessibility)	1b	2	3	4+ (Higher Accessibility)
Maximum dw (uha)	60	60	100	100	N/A

#### 3.1.3. Model Parameterization

**Figure 3:**
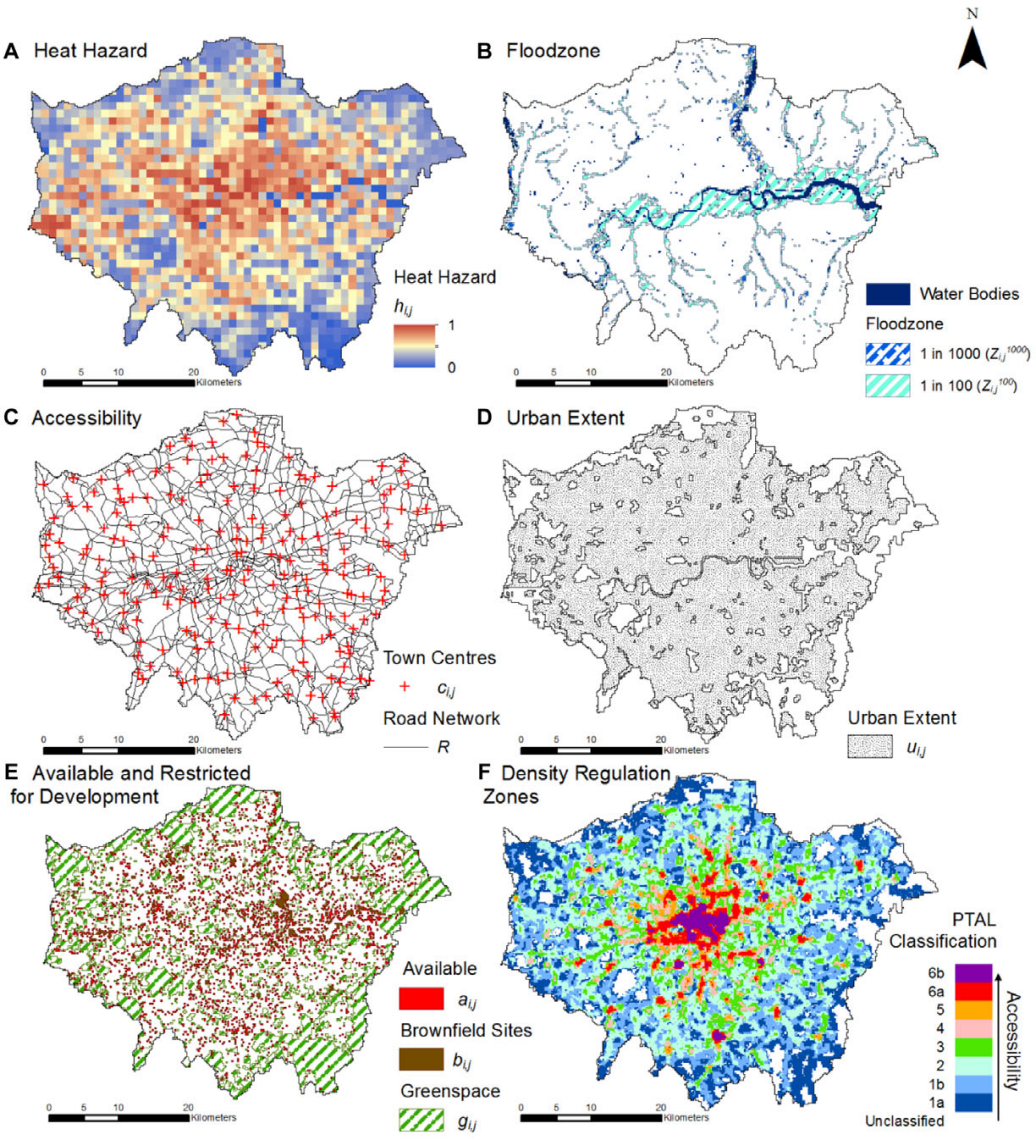
Spatial data sets for the case study (PTAL scale explained in Table I).

Fig. 3 presents the input data sets for the London application of the spatial optimization framework. Fig. 3(a) presents the spatial representation of heat hazard, $h_{ij}$, represented at 1 km spatial resolution by the UrbClim model.^55^ The model disaggregates an ensemble of IPCC climate change models then spatially models the effect of urban heat islands by using land cover data. Floodplain zones (Fig. 3b) were provided by the Environment Agency. The Ordnance Survey (OS, U.K. national mapping agency) Mastermap Strategi Settlement Seeds are used to represent London's town center network ($c_{ij}$) (Fig. 3c) while the road network, *R*, was extracted from the OS Meridian 2 roads data set. Fig. 3(d) shows the urban extents for the study area, $u_{ij}$, which were extracted and rasterized from OS Meridian 2 Developed Land Use Areas. Fig. 3(e) shows greenspace, $g_{ij}$, land potentially available for development,$\;a_{ij},$ and brownfield sites, $b_{ij}$, which are a subset of $a_{ij}$. Greenspace was defined as land in the OS Mastermap Topographic data defined as “Natural.” Areas available for development were all those in the OS Mastermap Topographic data that are not either developed or water bodies. Vector data for brownfield locations were provided by the London Development Agency's (LDA) London Brownfield Sites Database,^1^ before being rasterized to a 200 m spatial resolution. Of the 1,885 sites identified, the LDA's report found that 20% of the sites require remediation (8% full and 12% partial or potential).^67^ Finally, Fig. 3(f) shows the PTAL data set, which was also provided in vector format and rasterized to a 200 m grid (6b denotes the highest public transport accessibility while 1a denotes the lowest).

The GA parameters were selected on the basis of sensitivity testing carried out for an application of the spatial GA on a much smaller area (55 km^2^ as opposed to 1,572 km^2^ for London) that is detailed in Caparros‐Midwood.^68^ The smaller case study enabled exploration of the efficiency of the GA for different parameterizations that are summarized in Table II. These demonstrate simulations utilizing a larger parent set and fewer generations resulted in an average improved convergence in the objectives of 4%, but, more importantly, a significantly larger resulting MOPO set (see Section 2.3.2. for a definition of MOPO) with an average increase of 44%. This result is in line with findings in the literature.^69^ In addition, sources have stated the importance of large population for complex problems to ensure diversity.^70^ With this is in mind, and considering the increased complexity of the London planning problem, a parameterization comprising a higher ratio of No parents  compared to *G* was considered to be most appropriate. As recommended by Konak *et al*.,^58^ the total of *p*_crossover_ and *p*_mutation_ was set to 0.9 to ensure a small number of solutions (10%) are unchanged. As shown in Table II, values of 0.7 and 0.2 for *p*_crossover_ and *p*_mutation_, respectively, lead to improved convergence in the objectives (16% and 10% compared to the parameter sets) as well as increasing the total MOPO solutions found (12% compared to the nearest result) indicating that it enabled the most diverse set of solutions to be found. Based on these findings of the sensitivity testing, Table III outlines the simulation parameters utilized for the London application in this article. Due to the increased size (and therefore number of possible spatial development strategies) of the London case study, higher values of *G* and No parents  (listed in Table III) were required to ensure convergence of the algorithm.

**Table II:** Results of Sensitivity Testing Carried Out for the Spatial Optimization Framework (see Ref. 68)

	Average		
Parameter Value	Relative min(*f*_heat_)	Relative min (f dist )	No. of MOPO Solutions
G= 100	1.0	1.0	896
No parents = 1000			
G= 200	1.03	1.07	625
No parents = 500			
(Based on G= 50, No parents = 500)			
p crossover = 0.8	1.07	1.65	449
*p*_mutation_ = 0.1			
p crossover = 0.7	1.03	1.42	561
*p*_mutation_ = 0.2			
p crossover = 0.6	1.07	1.57	428
*p*_mutation_ = 0.3			

**Table III:** Run Parameters for Case Study Application of the Spatial Optimization Framework

Parameter	Description	Value
*G*	Number of generations	400
No parents	Number of parent *D* selected for each generation	2,500
*p*_crossover_	Probability of applying a crossover to two *D*	0.7
*p*_mutation_	Probability of applying a mutation to *D* within *offspring*	0.2
$p_{m}$	Probability of mutating an element ($d_{l}$) within *D*	0.05

### 3.2. Results and Discussion

**Figure 4:**
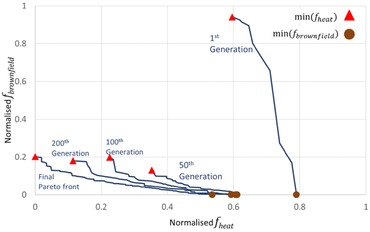
Convergence of the Pareto front (Pareto‐optimal set) between *f*_heat_ and *f*_brownfield_ throughout the GA operation.

Fig. 4 demonstrates the convergence of the Pareto front between *f*_heat_ and *f*_brownfield_ as well as the best performing spatial strategies for *f*_heat_ and *f*_brownfield_, min(*f*_heat_) and min(*f*_brownfield_), respectively, at stages of the GA operation. Within the first 50 generations there is a 86.2% improvement in *f*_brownfield_ for min(*f*_heat_) and a 22.78% improvement in *f*_heat_ for min(*f*_brownfield_). Thereafter, convergence slows with only a 11.3% improvement between the 50th and the final (400th) generation in *f*_heat_ for min(*f*_brownfield_) performance while *f*_brownfield_ for min(*f*_heat_) regresses 35% over the same time period to achieve the best found spatial strategy for *f*_heat_. Indeed, the number of Pareto‐optimal solutions between *f*_heat_ and *f*_brownfield_ increases from 9 in the initial generation to 115 by the final generation. Overall, the framework is able to improve the uptake of brownfield development by 78.7% from the first generation.

#### 3.2.1. Pareto‐Optimal Fronts

**Figure 5:**
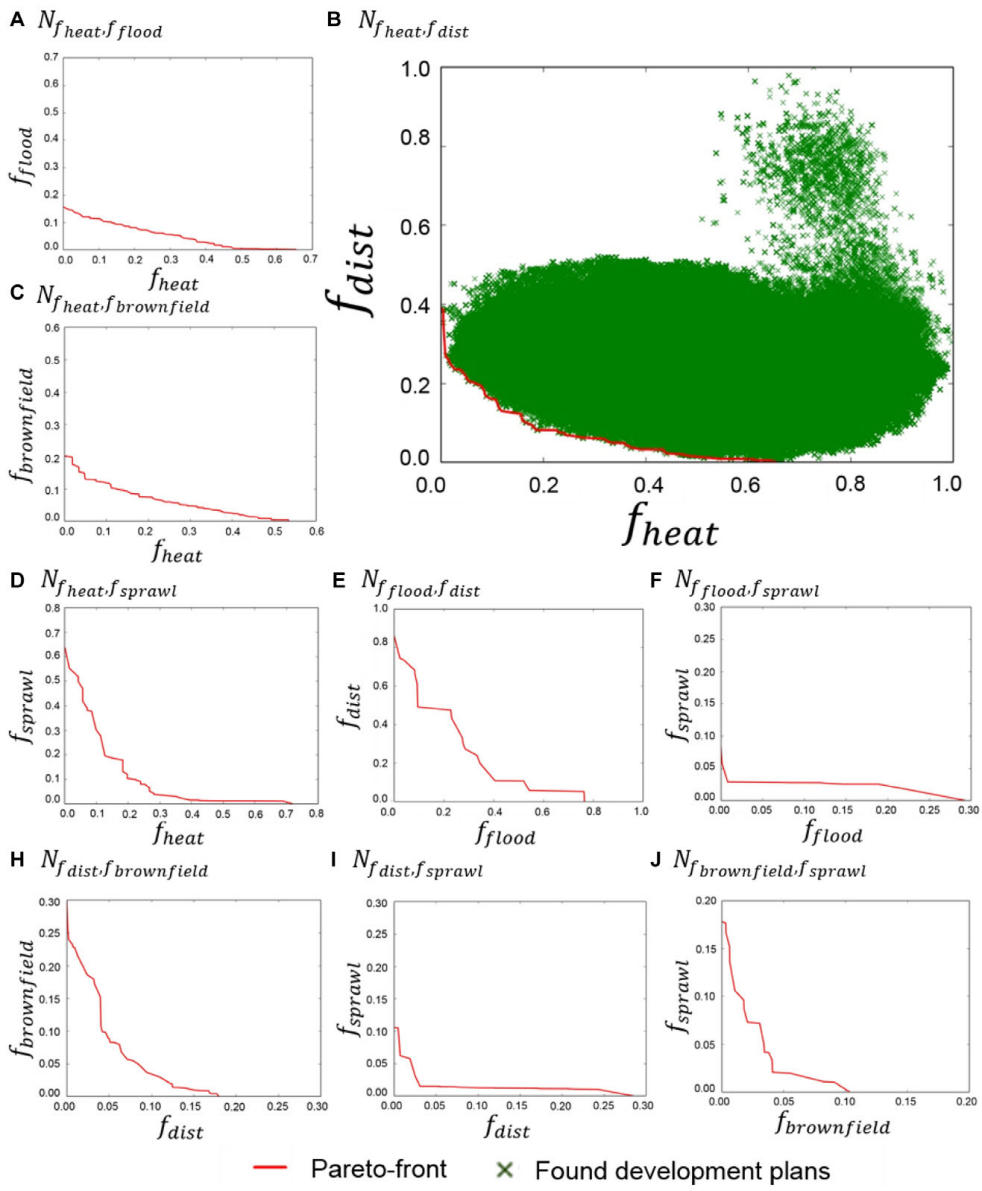
Normalized Pareto fronts between objectives optimized by the framework.

Fig. 5 presents the normalized performances of Pareto‐optimal fronts between pairs of objectives. Table IV quantifies the best tradeoffs between the objectives, and also provides the number of solutions that lie on each Pareto front. The results highlight clear conflicts between optimizing *f*_heat_ simultaneously with the other objectives (Figs. 5a–d). The solution min(f heat ) ⇒ f flood ≥0.16 while min(f flood ) ⇒ f heat ≥0.65 as areas next to the river with a low heat hazard are avoided. The spatial plans for min(*f*_dist_)⇒ f heat 0.65 and min(*f*_sprawl_) ⇒ f heat 0.72reflecting the increase in heat hazard close to high built‐up areas as a consequence of the land use. The best *f*_heat_ performance can be achieved with 85% of development on brownfield sites; however, to completely restrict development to brownfield the performance in min (f brownfield )⇒f heat ≥0.54. Conflicts between *f*_flood_ and the other objectives are much less pronounced with min(f flood )⇒f dist ≥0.08 and min(f sprawl )⇒f flood ≥0.12. The Pareto front between *f*_flood_ and *f*_brownfield_ is not shown as the framework is able to optimize both simultaneously, i.e.,  min (f flood ,f brownfield ).

Interestingly, *f*_dist_ and *f*_brownfield_ conflict significantly with min(f dist )⇒f brownfield ≥0.18 and  min (f brownfield )⇒f dist ≥0.3, suggesting a lack of brownfield sites in close proximity to town centers. A less intuitive result of the analysis is the conflict between *f*_dist_ and *f*_sprawl_ (Fig. 5i) with min(f dist )⇒f sprawl ≥0.29 and  min (f sprawl )⇒f dist ≥0.11 due to the proximity of some of London's town centers to the edge of the urban extent. Likewise, with *f*_brownfield_ and *f*_sprawl_ (Fig. 5j) to fully maximize one individual objective requires a significant tradeoff with the other. However, there are several spatial plans on their Pareto front that sacrifice limited performance in either objective for a better tradeoff, for example, f dist ≥0.03⇒f sprawl ge0.01.

**Table IV:** Pareto‐Front Trade‐Off Matrix

	Corresponding Value from the Pareto‐Front					
		*f*_heat_	*f*_flood_	*f*_dist_	*f*_brownfield_	*f*_sprawl_
Optimized objective: min().	*f*_heat_	NA	0.16 (113)	0.39 (64)	0.2 (115)	0.64 (55)
	*f*_flood_	0.65	NA	0.09 (20)	0 (1)	0.03 (11)
	*f*_dist_	0.65	0.08	NA	0.3 (44)	0.11 (27)
	*f*_brownfield_	0.54	0	0.18	NA	0.18 (21)
	*f*_sprawl_	0.72	0.12	0.29	0.1	NA
					(Number of solutions in Pareto front)	

#### 3.2.2. Pareto‐Optimal Spatial Configurations

**Figure 6:**
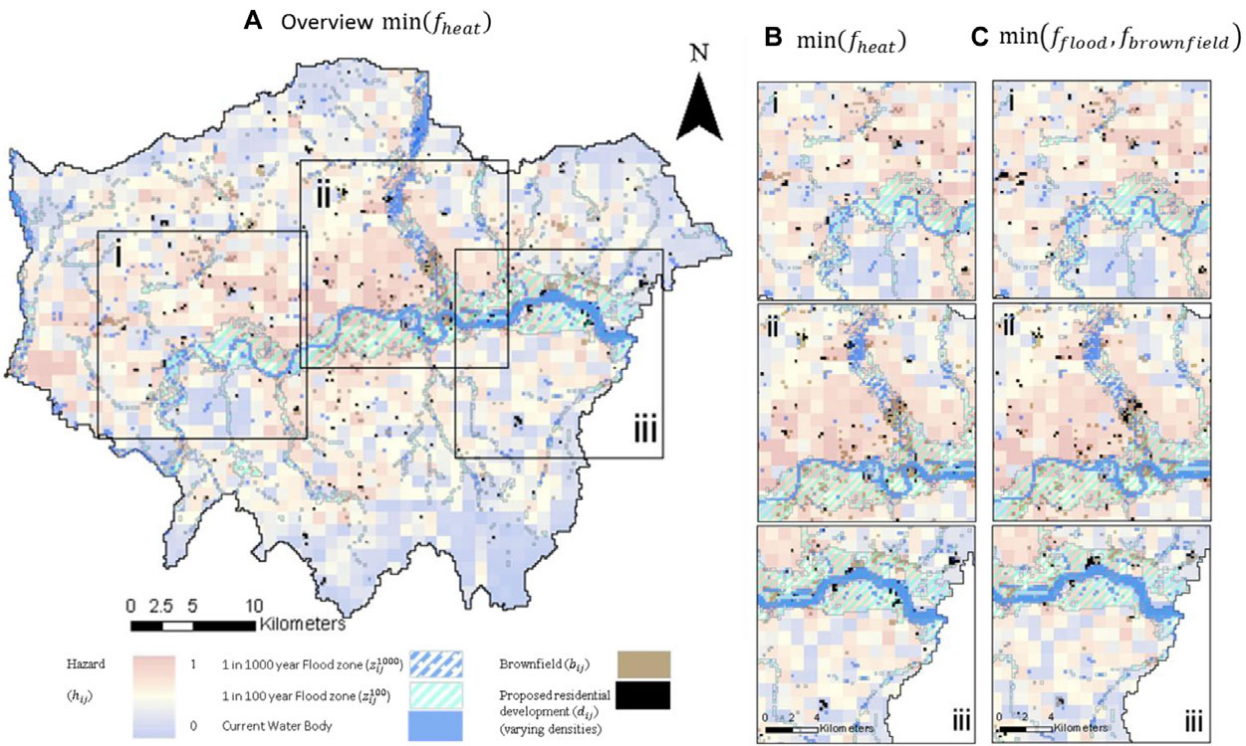
(a) Overview of spatial configuration for min(*f*_heat_), (b) viewing windows i, ii, and iii, and (c) comparison with spatial plan for  min (f flood ,f brownfield ). For clarity of visualization varied densities of development are not shown.

**Figure 7:**
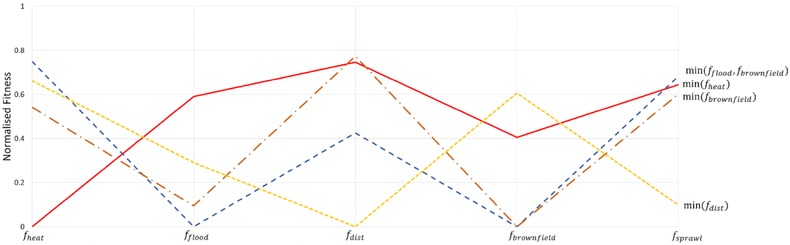
Parallel line plots for Pareto‐optimal spatial configurations against the objective outlined. Note that fitnesses are normalized throughout the MOPO set.

Fig. 6 presents the best spatial development strategy for min(*f*_heat_) as well as a comparison with the spatial configuration for  min (f flood ,f brownfield ) at highlighted areas. Fig. 7 provides a visual overview of the Pareto‐optimal solution's relative performance across the spectrum of objectives through a parallel line plot. The spatial configurations that are min(*f*_brownfield_) and min (f dist ) from the Pareto‐optimal sets Nf heat ,f brownfield and Nf heat ,fd ist ,respectively, are also shown to demonstrate the applicability of risk reduction with respect to current spatial planning procedures. Performances are normalized throughout the MOPO nondominated set.

Fig. 6(a) demonstrates how the spatial configuration strategically develops brownfield sites that correspond with lower heat hazard in order to achieve a best tradeoff with *f*_brownfield_ (the spatial configuration achieves a normalized performance of 0.41 within the MOPO set). Where possible these occur on the periphery of the study area. Indeed, several of these are consistent throughout the Pareto‐optimal spatial plans, signifying their suitability for development in the context of these pressures. However, in order to meet the dwelling target (Equation (1)) the strategy is forced to locate development centrally (Fig. 5b), which is where the greatest spatial variance occurs between strategies that are optimal for other criteria. Figs. 5(b) and 5(c) demonstrate how the strategies for min(*f*_heat_) and  min (f flood ,f brownfield ) vary spatially in these central areas. The spatial plan min(*f*_heat_) develops predominantly on the banks of the River Thames to take advantage of corresponding lower heat hazard. However, these correspond with floodzone causing a normalized performance of 0.6 in *f*_flood_ equating under this development strategy to 67,680 dwellings within the 1 in 100 floodzone and a further 17,200 within the 1 in 1,000 floodzone. While the spatial plan  min (f flood ,f brownfield ) avoids central London and concentrates on brownfield sites in the north and west of London, these correspond with higher heat hazard (reflected by the normalized performance of 0.75 in *f*_heat_).

Both development strategies min(*f*_heat_) and  min (f flood ,f brownfield ) perform poorly against *f*_sprawl_ (see Fig. 7), with 156 and 168 ha, respectively, of proposed development outside of current urban extent largely due to the development in the east of London (window iii). Conversely, Fig. 7 demonstrates how the development strategies min(*f*_brownfield_) and min (f dist ) perform poorly in *f*_heat_ as the assigned development occurs close to town centers and within the urban extent where heat hazard is higher. However, both perform relatively well in *f*_flood_ at 0.1 and 0.3, respectively.

#### 3.2.3. Comparison with Current Plans

**Figure 8:**
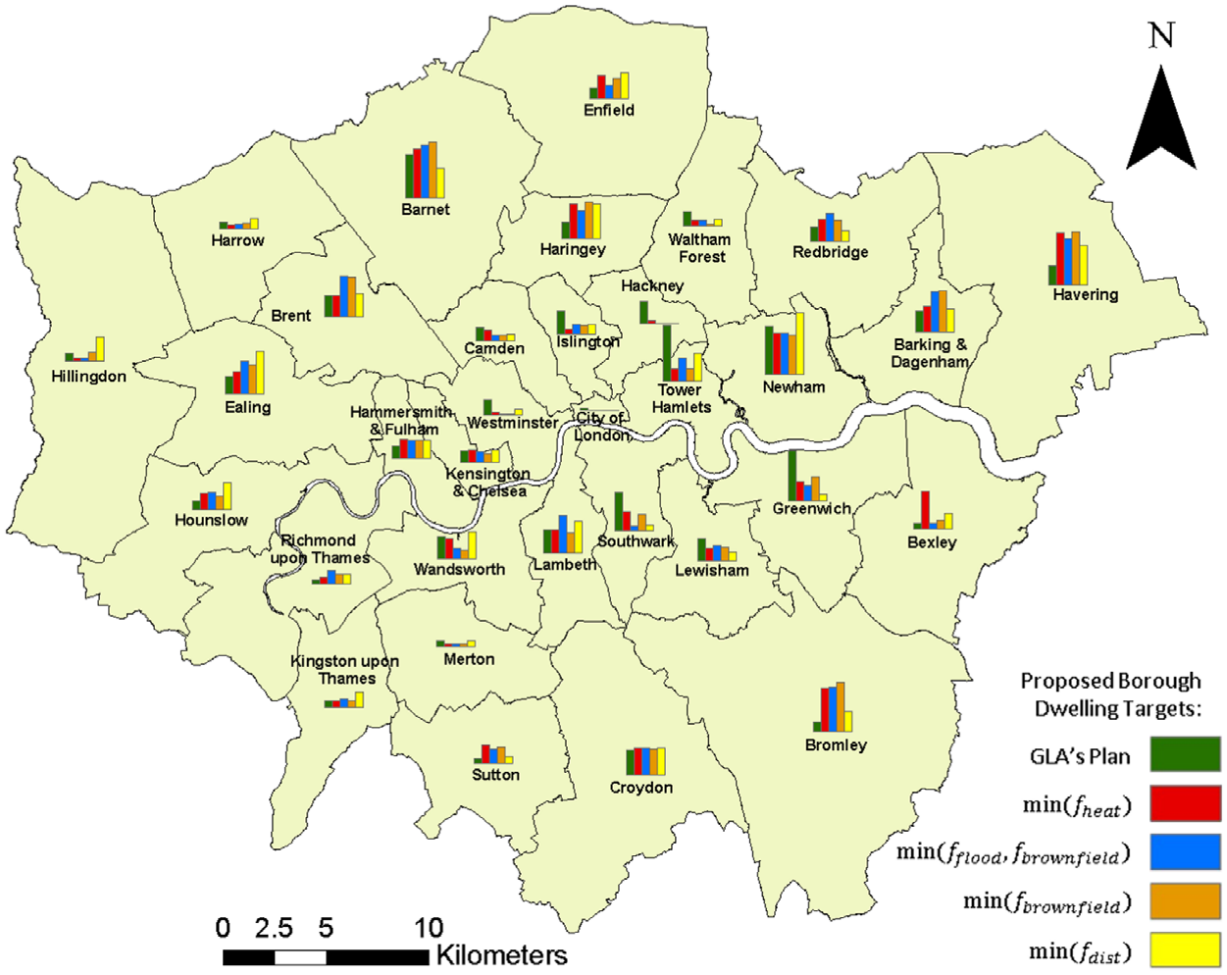
Comparison of London borough proposed dwelling totals based on Greater London Authorities and Pareto‐optimal solutions plans.

Fig. 8 presents a borough (local authority) scale comparison of spatial development between the GLA's spatial plan against the Pareto‐optimal spatial strategies. The comparison identifies several boroughs that are significantly more developed by all the Pareto‐optimal spatial plans, indicating that they are highly suitable development locations to meet a range of pressures while the current plans underutilize them. These include but are not exclusive to the more easterly and westerly boroughs of London. In addition, the analysis found the east London boroughs identified by the Greater London Spatial strategy to be assigned a large proportion of new development unsuitable to meet the prescribed risk and sustainability objectives, with found optimal development plans assigning consistently less development. For example, Hackney, with a GLA target of 11,600 dwellings by 2021, has close to no assigned development for the Pareto‐optimal spatial plans. Notably, several boroughs have similar numbers of assigned dwellings compared to the GLA's plan while multiple boroughs are highlighted for their suitability depending on the prioritization of the objectives. For example, Hillingdon has three times the number of dwellings assigned for min(*f*_dist_) compared to the GLA's plan while min(*f*_heat_) assigns nearly six times the number of dwellings proposed by the GLA to the Borough of Bexley. In comparison, Wandsworth has half the proposed dwellings for min(*f*_flood_) and min(*f*_brownfield_) assigned compared to the GLA's plan. These results together clearly demonstrate that the current plan is failing to meet risk and sustainability pressures by failing to optimize its performance with respect to the objectives outlined.

**Figure 9:**
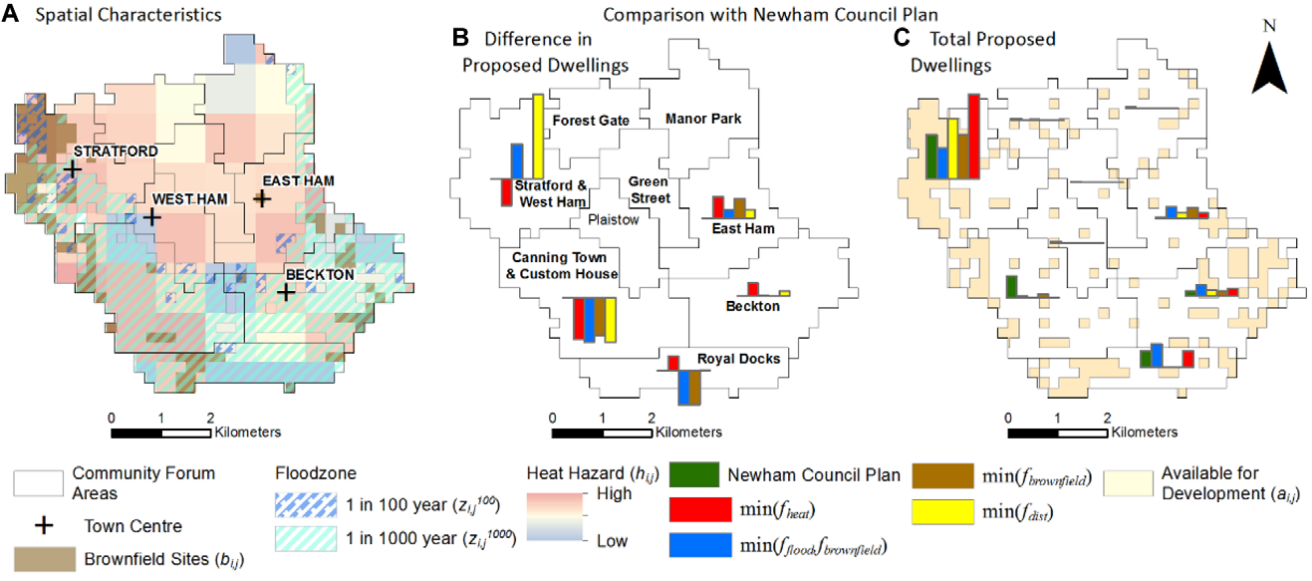
Comparison of the London borough of Newham's proposed dwelling totals based on Newham Council Development Strategy^(71)^ and Pareto‐optimal plans.

Fig. 9(a) details the spatial properties of the Borough of Newham (see Fig. 8 for location) while Figs. 9(b) and 9(c) present a subborough comparison of the difference in and total assigned development between the Newham Council Development Plan^71^ and the Pareto‐optimal plans, respectively. Due to the presence of multiple hazards and brownfield sites in the borough there is a lot of variance between the Pareto‐optimal plans. While Newham Council plans development in Canning Town and Custom House, the Pareto‐optimal spatial plan avoids these areas due to the presence of a floodzone and high heat hazard. Instead,  min (f flood ,f brownfield ) and min(*f*_dist_) concentrate development in the northwest of Newham and min(*f*_heat_) instead develops the east and southeast of Newham. Moreover, due to the presence of a floodzone and unsuitability of brownfield sites, both  min (f flood ,f brownfield ) and min(*f*_brownfield_) avoid the subborough zone of Royal Docks.

The spatial optimization approach sets out a series of tools for assessing the spatial impacts of differing trends of spatial development across a series of climate‐related and sustainability objectives. These can take the form of strategic assessments (such as Fig. 8) identifying the suitability of boroughs for development as well as lower planning scales such as administrative levels and wards (Fig. 9). The latter can help planners make better informed localized decisions to specific local challenges while contributing to overall risk management and sustainability planning. In addition, the analysis identifies trends that are optimal with regard to specific objectives as well as those that perform well over a combination of these. By providing planners with these optimal spatial trends the results can directly influence planning decisions.

## 4. CONCLUSIONS

In the presence of conflicting risk and sustainability pressures planners require decision support tools to better aid the balancing of priorities and allow for optimal planning decisions. In this article a spatial optimization framework has been developed to provide planners with a means of producing the evidence base for constructing spatial planning strategies that are optimal against multiple criteria and objectives. The work is unique in that it investigates the impact of development strategies over several sustainability elements simultaneously, namely, risk management, emission mitigation, and current planning policies, rather than an exclusive focus on individual sustainability themes. This approach is necessary to address full sustainability as experience has shown there are tensions between desirable objectives. In addition, the efficacy and applicability of the framework is demonstrated for a real‐world planning case study for a complex urban area, Greater London (U.K.), which covers a large area of 1,572 km^2^.

With these in mind, the results of the framework demonstrate its ability to produce optimal spatial development strategies that best balance the six risk and sustainability objectives investigated while also adhering to planning policies and land use constraints. Plans are found that are optimal against one or more of these objectives while diagnostic information from analysis of the results and Pareto sets, in particular, provides planners with detailed information on the magnitude and sensitivity of different tradeoffs between planning objectives. The case study also highlights the importance of spatial structure in modulating risks and other sustainability objectives: the different spatial structure of the flood and heat hazards limits the number of areas with low heat and flood risk, while the location of brownfield sites makes it impossible to exclusively develop these and optimize other objectives. In particular, the simultaneous examination of these differing sustainability goals allows for the discovery of a number of conflicts. Several of these conflicts are expected and corroborate the literature, such as that between accessibility and heat risk, and the framework works toward minimizing the conflict through strategic development strategies; for example, locating development in proximity to town centers in low heat risk areas. There is a common theme of conflicts between risks and other sustainability objectives, emphasizing the importance of considering risks alongside other planning objectives (see Figs. 5b–f). In addition, several conflicts are identified that are not as intuitive. The most significant of these with regard to the field of risk management is the conflict found between minimizing heat and flood risk.

Overall, the analysis finds that spatial strategies can be geared to optimally meet specific risk and sustainability objectives with regard to future development within London. However, it is not possible to simultaneously optimize all climate‐related hazards and sustainability objectives. Therefore, London, in terms of the spatial configuration of its potential future development, cannot maximize its full sustainability and resilience potential; instead, planners will need to prioritize a subset of objectives. Indeed, the analysis finds that different development strategies are needed to optimize development patterns that meet the two risk objectives, weakening the case that a city structure can provide resilience in its own right. Despite this, an approach such as the one presented in this article can identify development patterns that better deliver development priorities, while recognizing that some may only be achievable with social capacity building or demand management. With regard to London's spatial plan, the current strategy of significant development in east London is found to lead to a suboptimal performance in meeting the sustainability challenges investigated. However, these findings are limited to the objectives that are assessed in this work and are in the absence of other measures that can potentially alleviate conflicts between objectives (for example, building flood defenses). Thus, the consideration of future technologically‐driven adaption policies may well allow for an improved performance and potentially allow a city such as London to be both sustainable and resilient. Indeed, the work presented in this article provides an initial evidence base that can be further developed to help inform where and when adaption can be applied to move a city toward a sustainable and resilient future.

Further investigation is needed into the effect of assessing the cost of development strategies alongside their sustainability performances. This would reflect heterogeneities in land value across the city, but also explore the tension between the remediation of brownfield land at high cost and minimizing urban sprawl. While the work presented uses relatively simple metrics to evaluate risks and sustainability objectives, the framework is developed in such a way that more advanced risk calculations can easily be fitted into the evaluation phase of the framework shown in Fig. 1. For example, the flood risk calculation might be extended to include analysis of a wider range of flood return periods, or consideration of flood defense breach scenarios as described in Dawson and Hall.^72^ Moreover, and as already noted, the scope of the analysis could be improved by incorporating adaptation interventions, for example, the use of urban greening and/or building insulation, to assess their ability to improve the sustainability and resilience of the spatial allocation of development. However, more complex risk calculations and increased modeling need to be balanced with the resulting increased processing required, although this could potentially be offset by the use of cloud computing.

Lastly, many planning issues are ill suited to quantitative representation, and even if the spatial optimization framework were extended to consider a wider range of objectives the results should still be interpreted within the context of qualitative issues and used to improve the evidence base, rather than replace it in its entirety, for spatial planning.

## Footnotes

1
http://data.london.gov.uk/dataset/london-brownfield-sites-review

## APPENDIX 1 GLOSSARY OF TERMS

**Table A:** Notation Glossary

Problem Formulation	
Notation	Description
*d*	Proposed development site within *D*.
*D*	A proposed development plan consisting of proposed development sites $d_{l}$.
*l*	1, 2,…, *n*; where *n* is the total number of elements in *D*. Each *l* links to a ij location within London's study area via use of a lookup table.
i,j	A cell location within London's extent.
$a_{i,j}$	Cells identified as being available for development.
dens	Collection of possible development densities.
dw	Dwelling assigned to a cell based on proposed density and area of cell.
$D^{dw}$	Total number of dwellings associated with a proposed development plan, *D*.
Dw MAX	Maximum number of dwellings a feasible *D* can contain.
Dw MIN	Minimum number of dwellings a development plan, *D*, must contain.
**Parameterization**	
$h_{i,j}$	Heat wave hazard annual frequency raster.
$z_{i,j}^{1000}$	Cells within 1 in 1,000 floodzone.
$z_{i,j}^{100}$	Cells within 1 in 100 floodzone.
*C*	Collection of town centers centroids.
$c_{ij}$	Town center representing location of services and employment.
*R*	Road network.
*P*	Shortest path along the road network.
$b_{i,j}$	Cells designated as brownfield.
$u_{i,j}$	Cells designated as within the current urban extent.
$g_{i,j}$	Cells designated as greenspace.
**Pareto optimality**	
*f*	An element of *F*.
*F*	Set of objective functions.
*s*	A solution (spatial plan) found by the framework.
*N*	Nondominated list.
**Genetic algorithm**	
*G*	Number of generations.
No parents	Number of individuals to select for the next generation.
*p*_crossover_	Probability of applying a crossover to two individuals.
*p*_mutation_	Probability of mutating an individual.
$p_{m}$	Probability of mutating an element within an individual.
